# Does treating with anti–PD-1 to improve glomerular health come without a cost?

**DOI:** 10.1172/JCI164747

**Published:** 2022-11-15

**Authors:** Kenar D. Jhaveri, Abhijat Kitchlu, Ala Abudayyeh

**Affiliations:** 1 Glomerular Center at Northwell, Division of Kidney Diseases and Hypertension, Zucker School of Medicine at Hofstra/Northwell, Northwell Health, Great Neck, New York, USA.; 2 Department of Medicine, Division of Nephrology, University Health Network, University of Toronto, Toronto, Canada.; 3 Division of Internal Medicine, Section of Nephrology, University of Texas MD Anderson Cancer Center, Houston, Texas, USA.

**Keywords:** Nephrology, Cancer immunotherapy, Chronic kidney disease

## To the Editor:

We read with great interest the study by Pippin et al. (1) that highlighted the anti-aging effect of the podocyte with anti–programmed cell 1 death (anti–PD-1) antibodies. In addition, the authors elegantly showed that administration of anti–PD-1 antibody treatment to young mice with experimental focal segmental glomerulosclerosis (FSGS) lowered proteinuria and improved total podocyte counts.

**Figure 1:**
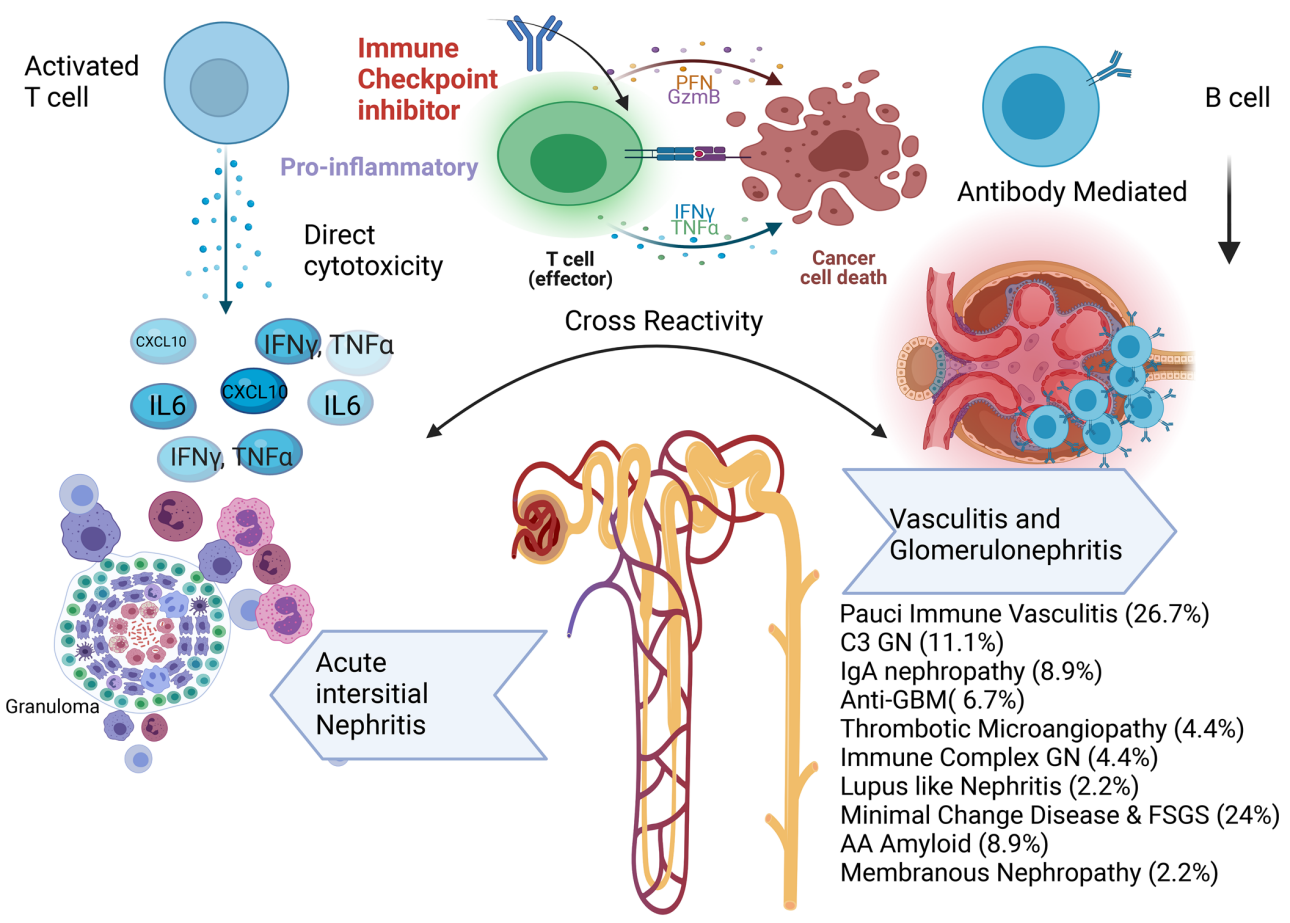
Spectrum of kidney injury associated with ICIs with a focus on glomerular-associated diseases. Anti-GBM, anti-glomerular basement membrane disease. Glomerular disease data are from Kitchlu et al. (3). Figure created using BioRender.

Clinically, as practicing onco-nephrologists, we have seen patients with use of immune checkpoint inhibitors (ICIs), specifically anti–PD-1 agents, and new-onset glomerular disease. In general, the incidence of acute kidney injury (AKI) via kidney immune-related adverse events was 2% to 3% in a large multicenter study (1, 2). Acute interstitial nephritis has been reported as the most common kidney biopsy finding, although many glomerular lesions have been reported. Pauci-immune vasculitis, podocytopathies (including minimal change disease and FSGS), and C3 glomerulonephritis (GN) have been the most common glomerular diseases described. In an analysis of all published case reports and case series of glomerular pathology findings (3) associated with ICIs, 45 cases of biopsy-confirmed ICI-associated glomerular disease were identified. Several lesion types were observed, with the most frequent being pauci-immune GN and renal vasculitis (27%), podocytopathies (24%), and complement 3 GN (C3GN) (11%) (Figure 1). The World Health Organization–Uppsala Monitoring Center Causality Assessment System was used to assess the likelihood of a causal relationship between ICI and glomerular disease, with 40 of 45 cases receiving a category of “probable” or higher. Concomitant acute interstitial nephritis was reported in 41%. Most patients had ICIs discontinued (88%), and nearly all received corticosteroid treatment (98%). Complete or partial remission of proteinuria was achieved in 45% and 38%, respectively. Renal replacement therapy (RRT) was required in 25%. Most patients had full (31%) or partial (42%) recovery from AKI, although 19% remained dialysis dependent and approximately one-third died. Since then, several other glomerular pathologies have been reported and observed (our unpublished observations) in clinical practice — such as membranous nephropathy and other podocytopathies (4) (Figure 1).

The pathophysiology linking ICI use and these multiple forms of glomerular pathology remains unclear, but these glomerular diseases observed thus far portend poor kidney and cancer outcomes in many cases. The authors rightfully discuss tubulointerstitial nephritis and glomerular disease that are associated with ICI use in cancer patients and explain that in their study, podocytes were preserved with no evidence of effacement. This indicates the lack of autoimmune induction and further supports that the use of anti–PD-1 antibody in mice improves podocyte life span. One can argue that continuous exposure to anti–PD-1 can provide initial benefit, but long-term use can lead to autoimmune activation, where median time to GN from time of exposure of ICI in cancer patients was noted to be 13 weeks (3), while in the current study mice were evaluated at 4 and 8 weeks after treatment with anti–PD-1. Further support of the autoimmune etiology of disease induction is provided by the successful use of rituximab for treatment of ICI-induced vasculitis (5).

While we applaud the authors on their experiments in animal models, we would advise caution in practice regarding the use of ICIs in treatment of podocytopathies and other glomerulopathies.

## Version 1 11/15/2022

Electronic publication
